# Adipose tissue in COVID-19: detection of SARS-CoV-2 in adipocytes and activation of the interferon-alpha response

**DOI:** 10.1007/s40618-022-01742-5

**Published:** 2022-02-15

**Authors:** A. Basolo, A. M. Poma, D. Bonuccelli, A. Proietti, E. Macerola, C. Ugolini, L. Torregrossa, R. Giannini, P. Vignali, F. Basolo, F. Santini, A. Toniolo

**Affiliations:** 1 Obesity and Lipodystrophy Center, Endocrinology Unit, University Hospital of Pisa, 56124 Pisa, Italy; 2 Department of Surgical, Medical, Molecular Pathology and Critical Area, University Hospital of Pisa, Pisa, Italy; 3 Department of Forensic Medicine, Azienda USL Toscana Nordovest, Lucca, Italy; 4 Global Virus Network, University of Insubria, 21100 Varese, Italy

**Keywords:** COVID-19, Pathogenesis, Virus, Immune transcriptome, Interferon, BMI, Autopsy

## Abstract

**Objective:**

Obesity is a recognized risk factor for the progression to severe forms of COVID-19, yet the mechanisms of the association are unclear.

**Methods:**

Subcutaneous abdominal adipose tissue specimens of subjects deceased from COVID-19 (*n* = 23) were compared to those of controls dying abruptly from causes other than infectious (accidental trauma, sudden cardiac death). Alterations of lung parenchyma consistent with moderate to severe disease were detected in all COVID-19 cases, not in controls. Investigations included: histopathologic features, detection of virus antigens and genome, characterization of infiltrating leukocytes, transcription levels of immune-related genes.

**Results:**

By RT-PCR, the SARS-CoV-2 genome was detected in the adipose tissue of 13/23 (56%) cases of the COVID-19 cohort. The virus nucleocapsid antigen was detected in the cytoplasm of 1–5% adipocytes in 12/12 COVID-19 cases that were virus-positive by PCR in the adipose tissue (one case could not be assessed due insufficient tissue). The adipose tissue of COVID-19 cases showed leukocyte infiltrates and upregulation of the interferon-alpha pathway. After adjusting for age and sex, the activation score of IFN-alpha was directly related with transcription levels of the ACE2 gene, a key entry factor of SARS-CoV-2.

**Conclusions:**

In lethal COVID-19 cases, the SARS-CoV-2 nucleocapsid antigen has been detected in a sizeable proportion of adipocytes, showing that the virus may directly infect the parenchymal cells of subcutaneous fat. Infection appears to activate the IFN alpha pathway and to attract infiltrating leukocytes. Due to the huge numbers of adipocytes in adults, the adipose tissue represents a significant reservoir for SARS-CoV-2 and an important source of inflammatory mediators.

## Introduction

The white adipose tissue (WAT) communicates with other tissues to regulate metabolism both centrally and peripherally through secretion of adipocyte-derived peptide hormones, inflammatory mediators and signaling lipids [1–3]. WAT is an essential regulator of energy storage and systemic metabolic homeostasis. One of its endocrine functions is the conversion of androstenedione to estrone, the major source of estrogen in men and post-menopausal women [4]. In addition to releasing regulators of glucose and lipid metabolism, WAT cells (adipocytes, stromal cells, resident innate lymphoid cells, dendritic cells, monocyte-derived macrophages) produce hormones (e.g., leptin, adiponectin, resistin, apelin), growth factors (e.g., FGF21, BMPs, TGF-beta, VEGFs, PDGF) and cytokines (e.g., TNF, IL6, IL13, CCL2)[1, 2].

Obesity has been recognized as a risk factor for progression to severe forms of COVID-19 [5]. The mechanisms underlying the link between obesity and disease severity upon infection with SARS-CoV-2 remain, however, unclear. In principle, obesity could contribute in multiple ways to the evolution of infection: (a) down-modulating the antiviral responses [6]; (b) releasing lipids that promote endothelial dysfunction and sustain intravascular coagulation [7]; (c) disrupting leptin and insulin signaling, thus intensifying the inflammatory response [8]; (d) promoting enhanced expression of SARS-CoV-2 receptors [9]; (e) representing a large reservoir for virus replication with increased shedding of virus and inflammatory mediators [10].

Recently, Gao and colleagues [11] evaluated the risk of severe COVID-19 outcomes in patients with obesity. The findings showed a positive association between body mass index (BMI) and COVID-19 hospital admissions and deaths. For hospital admissions, risk began to increase linearly above a BMI of 23 kg/m^2^, whereas the risk of death began to increase at slightly higher BMI of 28 kg/m^2^, in keeping with the association between BMI and a wide range of cause-specific mortality outcomes [12]. SARS-CoV-2 was detected in multiple endocrine tissues such as thyroid [13–15] and testis of subjects dying from COVID-19 [16]. In addition, a recent study has shown the association of high body mass index with an increased risk of developing COVID-19 [17].

Due to the increasing prevalence of obesity in developed countries [2, 18], we aimed at investigating the effects of SARS-CoV-2 in abdominal fat of individuals who died from COVID-19. Autopsy specimens of subcutaneous WAT of 23 COVID-19 autopsy cases were compared to specimens of 12 cases of sudden death from non-infectious causes. Results show that SARS-CoV-2 antigens are expressed into adipocytes of COVID-19 cases, that inflammatory infiltrates are well represented, and that transcription of the angiotensin-converting enzyme-2 (ACE2) key entry factor for the virus is upregulated in the infected adipose tissue. In addition, mRNA transcription of selected genes of the innate antiviral response is enhanced.

## Materials and methods

### Investigated cases and study design

As shown in Table 1, three case groups have been investigated: (a) controls, i.e., subjects who died from acute causes other than infectious (trauma, sudden cardiac death; *n* = 12); (b) patients dying from COVID-19 (abdominal adipose tissue negative for SARS-CoV-2; n = 10); (b) patients dying from COVID-19 (abdominal adipose tissue positive for SARS-CoV-2; *n* = 13). Results refer to subjects of Caucasian ethnicity. Autopsies have been performed at the Unit of Forensic Medicine, Azienda USL Toscana Nordovest, Lucca, Italy. The recruitment area of the Institution includes four major City Hospitals: Lucca, Pisa and Massa-Carrara caring for approximately 1.3 million populations.

**Table 1:** Demographic, clinical and virological features of the investigated autopsy cases

Features	Controls (*n* = 12)	COVID-19 patients (*n* = 23)	
	SARS-CoV-2-negative (*n* = 12)	SARS-CoV-2-negative in adipose tissue (*n* = 10)	SARS-CoV-2-positive in adipose tissue (*n* = 13)
*Detection of SARS-CoV-2 by RT-PCR*			
Lungs	–	+	+
Adipose tissue	–	–	+
*Age (years)*			
Mean (SD)	57.2 (20.3)	64.5 (19.2)	74 (14.6)
*Gender*			
Male	8 (67%)	5 (50%)	11 (85%)
Female	4 (33%)	5 (50%)	2 (15%)
*BMI*			
Normal	9 (75%)	2 (20%)	6 (46%)
Overweight/obesity	3 (25%)	8 (80%)	7 (54%)
*Comorbidities*			
Cardiovascular disease	6 (50%)	4 (40%)	8 (62%)
Chronic pulmonary disease	0	3 (30%)	1 (8%)
Diabetes	1 (8%)	2 (20%)	4 (31%)
Malignancy	0	1 (10%)	1 (8%)
Hypokinesia	0	0	1 (8%)
None	5 (42%)	4 (40%)	3 (23%)
*Days from COVID-19 symptoms to death*			
Mean (± SD)	N.A	15.9 (17)	7.2 (5.4)*

^*^After adjusting for age, sex and BMI, Cox regression showed that SARS-CoV-2 positivity of subcutaneous WAT is associated with a reduced survival time from initial symptoms (HR 3.7, 95%CI 1.1–12.5, *p* = 0.03)

*BMI* body mass index (kg/m^2^)

**Fig. 1:**
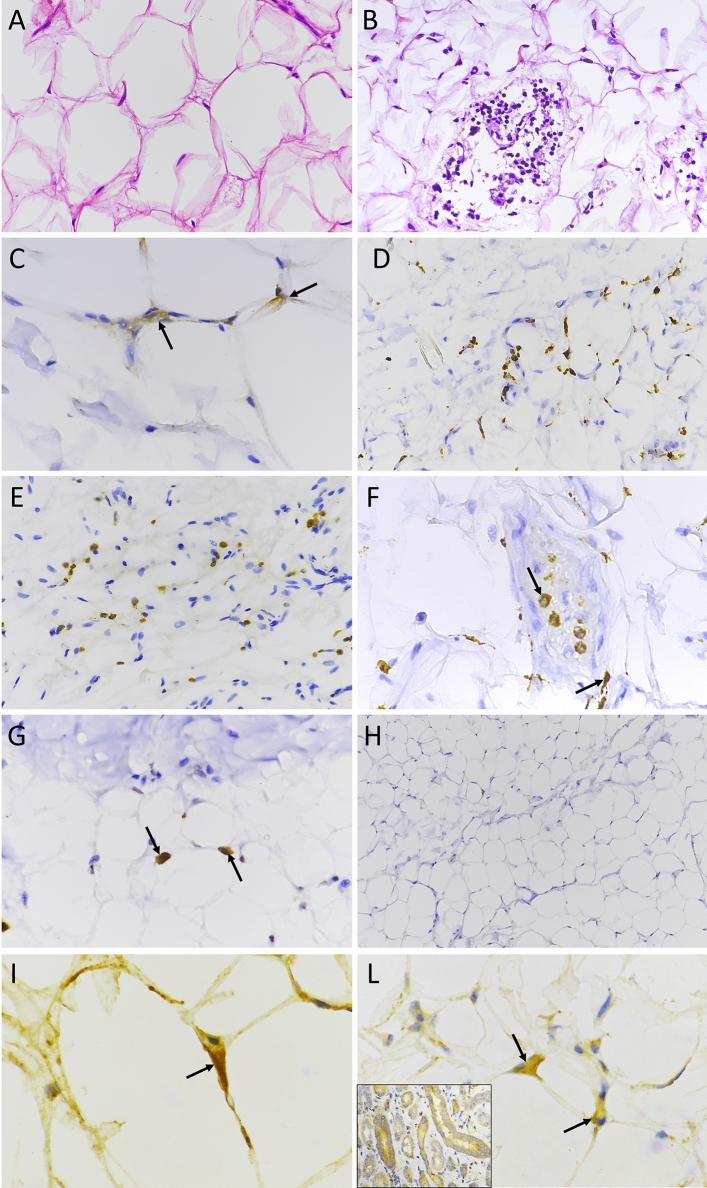
Histopathology and immunostaining of subcutaneous adipose tissue of COVID-19 autopsy cases. **A** H&E staining of adipose tissue from a control case (40X); **B** H&E staining showing leucocyte infiltration in a COVID-19 case positive for SARS-CoV-2 in adipose tissue (40X). Immunostaining: **C** nucleocapsid protein in the cytoplasm of two adipocytes (black arrows; 60X); **D** staining for pan-leukocyte marker CD45 (40X); **E** staining for the CD3 marker shows T-cells infiltrating the tissue (40X); **F** staining for CD68: black arrows show macrophage infiltration (60X). **G** staining for CD57: black arrows show natural killer cells (60X); **H** absence of staining for CD20 indicates the lack of infiltrating B-cells in SARS-CoV-2 positive adipose tissue (20X); **I** and **L** expression of the interferon-induced PKR protein in the cytoplasm of adipocytes of two different COVID-19 cases (60X); L, inset) PKR-positive control: marked PKR staining in the cytoplasm of tubular cells in normal human kidney

At autopsy, all cases were screened for the SARS-CoV-2 genome in the inferior lobe of both lungs as well as in the abdominal adipose tissue. As previously reported [14], alterations of lung parenchyma consistent with moderate to severe disease were detected in all COVID-19 cases, not in controls. Adipose tissue specimens of COVID-19 cases were compared to those of controls with regard to histopathological features (Fig. 1A and B), transcription levels of immune-related genes, infiltrating leukocyte markers, expression of SARS-CoV-2 antigens, and detection of SARS-CoV-2 genome by gene amplification. The study was approved by the local Ethical Committee (Comitato Etico Area Vasta Nord-Ovest, Italy No. 17327, 2020–05-14).

### Detection of viral genome and analysis of gene transcription

For each specimen, total RNA was extracted from four 10 µm-thick FFPE sections of abdominal WAT using the RNeasy FFPE kit (Qiagen, Hilden, Germany) and quantified by spectrophotometry (Trinean, Gentbrugge, Belgium). About 250 ng of RNA were used for each RT-PCR test to detect two genes of SARS-CoV-2 [viral nucleocapsid (N) and RNA-dependent RNA Polymerase (RdRp)] using the SARS-CoV-2 WE kit (Diatech Pharmacogenetics, Jesi, Italy). All samples were run in duplicate. Results were deemed positive when amplicons were obtained from at least one of the two genes. To evaluate the expression of viral nucleocapsid antigens and PKR by immunohistochemistry (IHC), 30 randomly selected high power (40x) fields were scanned, and the percentage of stained cells was calculated.

Gene expression analysis was performed by the nCounter technology (nanoString Technologies, Seattle, WA, USA) using two panels: (a) the Host Immune Response panel; (b) the Coronavirus panel plus that contains probes targeting the N and S ORFs of HCoV-229E, HCoV-HKU1, HCoV-NL63, HCoV-OC43, SARS-CoV, and the ACE2 virus receptor. Probes for the SARS-CoV-2 virus were designed according to the reference sequence, Wuhan-Hu-1 (NC_045512). For the assay, about 250 ng of RNA were hybridized with probes at 65 °C for 21 h. Procedures were performed following the manufacturer’s protocol.

### Immunohistochemistry

Immunostaining for viral proteins was performed in all cases except for one case that was not assessed due insufficient tissue. Markers of immune cells have been determined in 11/12 controls, 7/10 COVID-19 cases virus-negative in WAT, 11/13 COVID-19 cases virus-positive in WAT. In some cases, the analysis could not be performed due to insufficient material. Three-micrometer-thick sections were stained with antibodies to SARS-CoV-2: rabbit polyclonal antibody to the nucleocapsid protein (NB100-56,683, Novus Biologicals, Centennial, CO, United States) and mouse monoclonal antibody to SARS-CoV-2 spike glycoprotein (GTX632304, GeneTex). Antibodies to immune cell markers (Roche Diagnostics, Ventana Medical Systems, Oro Valley, AZ, United States) comprised: CONFIRM anti-CD3 (2GV6) rabbit monoclonal, CONFIRM anti-CD20 (L26) mouse monoclonal, CONFIRM anti-CD45, LCA (RP2/18) mouse monoclonal, CONFIRM anti-CD68 (KP-1) mouse monoclonal, anti-CD57 (NK1) mouse monoclonal, CONFIRM anti-CD15 (MMA) mouse monoclonal. Staining for the human RNA-dependent protein kinase (PKR) was performed using a PKR rabbit polyclonal antibody (Invitrogen-ThermoFisher Scientific; 1:50 dilution, incubation at 36 °C for 48 min). Sections of normal human kidney were used as positive control [19]. Staining procedures were performed with an automated staining system (BenchMark ULTRA, Ventana Medical Systems). Antibody binding was revealed with the OptiView DAB IHC Detection Kit (Ventana Medical Systems). Slides were counterstained with Hematoxylin II and Bluing Reagent (Ventana Medical Systems). Counts of immune markers were performed independently by two pathologists (A.P. and F.B). Twenty randomly selected fields were scanned at 20 × magnification. IHC score: cell counts represent the mean numbers of cells per square mm.

### Data analysis

Raw data of gene transcripts were normalized using the Advanced Analysis module of the nSolver v.4.0 (nanoString Technologies). Low count genes defined as those with raw expression level as low as 20 counts were omitted. Normalized gene expression levels were log2 transformed for further analysis. The principal component analysis (PCA) was performed by the procedures of the PCAtools Bioconductor package v.3.12 after removing 10% of genes with the lowest variance. Differentially expressed genes (DEG) were computed by a linear model adjusting for age, sex and BMI, and using the Advanced Analysis module of nSolver. In detail, control cases were used as baseline and three comparisons were done: all COVID-19 cases *vs* baseline; COVID-19 cases with no virus detected in WAT *vs* baseline; COVID-19 cases with SARS-CoV-2 detected in WAT *vs* baseline.

The IFNα score was computed by single sample Gene Set Enrichment Analysis (ssGSEA) using the procedures of the GSVA Bioconductor package v.1.38.2. The method described by Barbie [20]  was used. Gene Set Enrichment Analysis (GSEA) was run using the ranked gene list of differential expression analyses and following the procedures of the clusterProfiler Bioconductor package v.3.18.1. For analysis, ten was used as minimum gene set size cut-off, and the Hallmark collection of the Molecular Signatures Database (MSigDB) v.7.4 behaved as a reference [21] . Gene functions are reported according to the human gene databases GenCards and OMIM.

Survival times after initial symptoms were analyzed by Cox regression both in univariate and multivariate setting following the procedures of the survival R package v.3.2–13. Linear regression analysis was performed to evaluate the relationships among BMI and transcripts for SARS-CoV-2 entry molecules (ACE2, TMPRSS2, FURIN), as well as those between BMI and IFN alpha score. Pearson’s correlation coefficient was then calculated to quantify the associations of BMI and IFN-alpha score after adjusting for age and sex. Scatter plot were generated after partial regression. Analyses and plots were generated in R environment (https://www.r-project.org/, v.4.0.2, last accessed May 10, 2021) and with Prism (GraphPad Software, San Diego, CA).

## Results

### Nucleocapsid antigen of SARS-CoV-2

The demographic and clinical data of the investigated autopsy groups are shown in Table 1. Compared to controls (sudden death from trauma or sudden cardiac arrest), overweight or obesity were more frequent among COVID-19 cases (*p* = 0.03). All COVID-19 patients were positive for the SARS-CoV-2 genome in lungs. Among COVID-19 cases, however, the virus genome was detected in abdominal WAT of 13/23 (56%) subjects. Uninfected controls and COVID-19 cases whose WAT was SARS-CoV-2-negative by PCR were not stained by antibodies to the virus nucleocapsid antigen. Conversely, 12/12 COVID-19 cases whose WAT was virus-positive by PCR showed granular cytoplasmic staining in 1–5% adipocytes. Due to insufficient material, one SARS-CoV-2-positive case by PCR could not be assessed by IHC (Fig. 1C). In the same specimens, the antibody to the SARS-CoV-2 spike glycoprotein produced diffused staining that did not allow the precise localization of the viral protein (data not shown).

The nCounter system—based on the direct hybridization of probes without amplification steps—could detect the virus genome in 7/13 (54%) WAT specimens of COVID-19 cases that were virus-positive by PCR in abdominal fat. In the latter cases, hybridization occurred with probes targeting SARS-CoV-2, not with probes specific for other coronaviruses (HCoV-229E, HCoV-HKU1, HCoV-NL63, HCoV-OC43, SARS-CoV). As expected, hybridization failed to detect coronaviruses in WAT of controls and of COVID-19 cases whose WAT was virus-negative by PCR.

Finally, it was evaluated whether SARS-CoV-2 positivity in subcutaneous WAT impacted survival times. Using a univariate setting, the association was not statistically significant (*p* = 0.13). However, upon adjustment for age, sex and BMI, detection of SARS-CoV-2 in subcutaneous WAT was associated with a reduced survival time from initial symptoms (HR 3.7, 95%CI 1.1–12.5, *p* = 0.03).

### Leukocyte infiltrates and expression of RNA-dependent protein kinase (PKR) as a marker of IFN activation

**Fig. 2:**
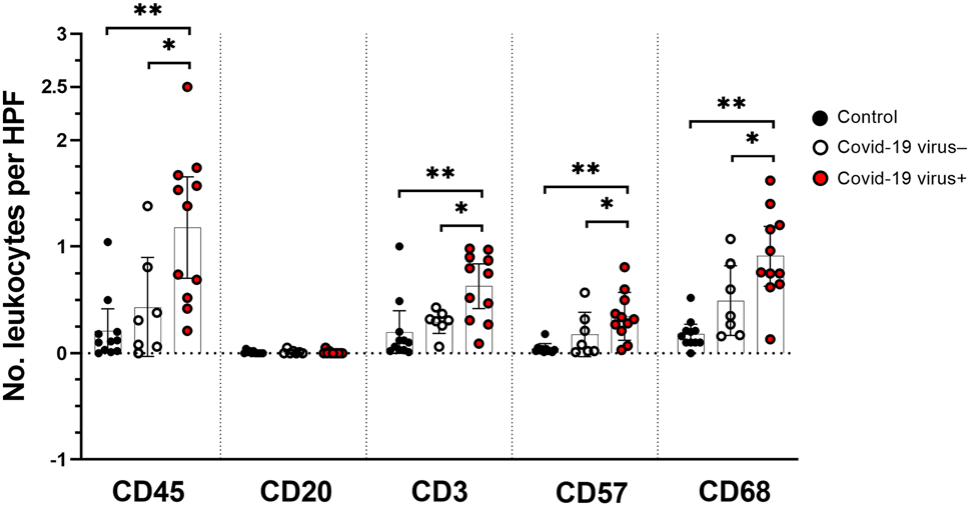
Numbers of leukocytes per high power field (HPF) as evaluated by immunohistochemistry in subcutaneous adipose tissue. IHC scores for the following markers: CD45 (pan-leukocyte), CD20 (B cells), CD3 (T cells), CD57 (NK cells), CD68 (macrophages). Vertical black lines show interquartile ranges; horizontal black lines indicate the median value. COVID-19 cases: virus-positive specimens of adipose tissue (*n* = 11, red dots), virus-negative specimens of adipose tissue (*n* = 7, white dots). Control cases (*n* = 11, black dots). Counts represent the mean leukocyte numbers per square mm. In a few cases, leukocyte markers could not be determined due to insufficient material. *p < 0.05 (case vs. the indicated group; Dunn’s test for multiple comparisons). **p < 0.001 (case vs. the indicated group; Dunn’s test for multiple comparisons)

Inflammatory infiltrates were evaluated by staining for leukocyte markers. Compared to controls and to COVID-19 specimens that were negative for virus in WAT, virus-positive specimens showed significantly increased numbers of cells expressing CD45 pan-leukocyte marker (*p* < 0.01) (Fig. 1D), CD3 T-cells (*p* < 0.05) (Fig. 1E), CD57 natural killer cells (*p* < 0.05) (Fig. 1F), and CD68 macrophages (*p* < 0.05) (Fig. 1G) (Fig. 2). CD20 B-cell leukocytes could not be detected in any groups (Fig. 1H).

Expression of the interferon-induced PKR protein (whose activated form phosphorylates the Eukaryotic Translation Initiation Factor 2 Subunit Alpha (EIF2S1) which, in turn, inhibits protein synthesis) was evaluated by immunostaining [19]. Compared to the control group, WAT specimens of all COVID-19 cases showed significantly increased numbers of adipocytes expressing PKR, no matter if WAT specimens were virus-positive or virus-negative (Fig. 1I, L). In COVID-19 cases, each high-power field (40x) comprised 2–6 PKR-positive adipocytes. This was significantly different (*P* < 0.05) from what observed in controls which had less than 0.3 PKR-positive cells per high power field (data not shown).

Sections of normal human kidney were used as positive control for PKR staining. As expected, PKR was highly expressed in the cytoplasm of kidney tubular cells (Fig. 1 L, inset).

### Gene expression analysis

**Fig. 3:**
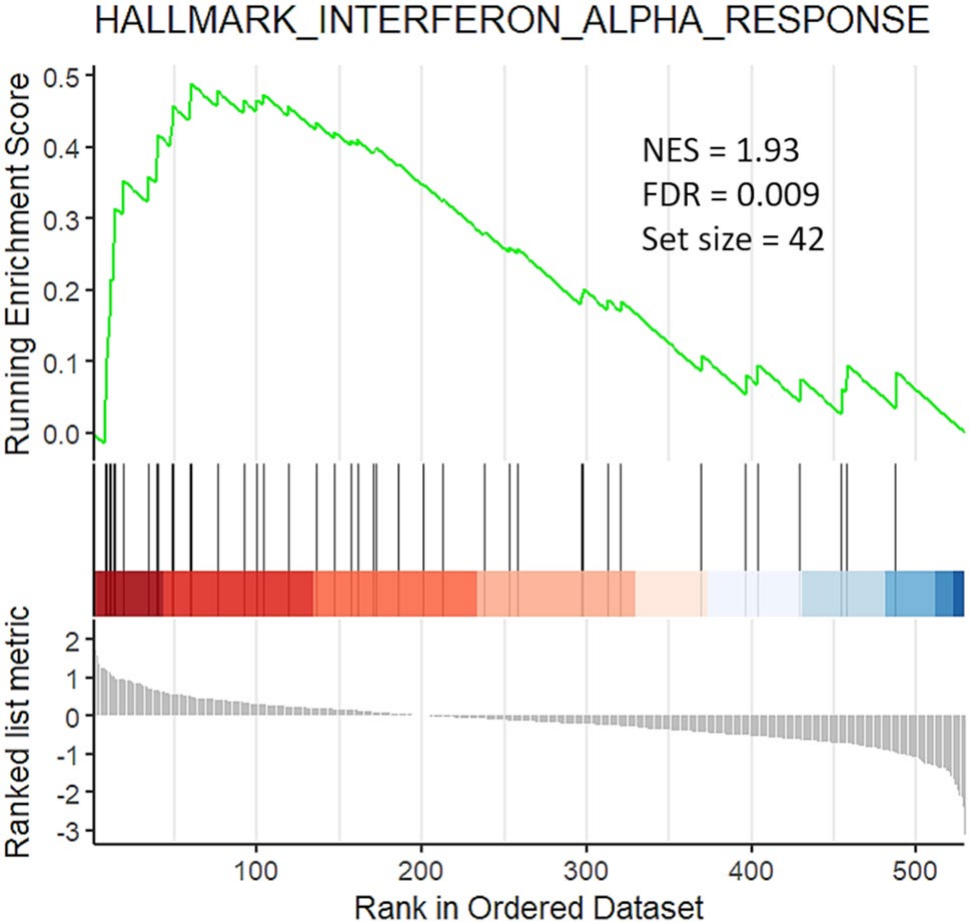
Gene set enrichment analysis plot of adipose tissue specimens of COVID-19 cases vs. control cases. Compared to the control group (*n* = 12), the IFN-alpha pathway was activated in COVID-19 cases that were SARS-CoV-2-positive by PCR in adipose tissue (13 cases). Vertical black bars refer to genes activated upon IFN-alpha signaling and are shifted to the left indicating a positive fold change. Abbreviations: NES, normalized enrichment score; FDR, false discovery rate

**Fig. 4:**
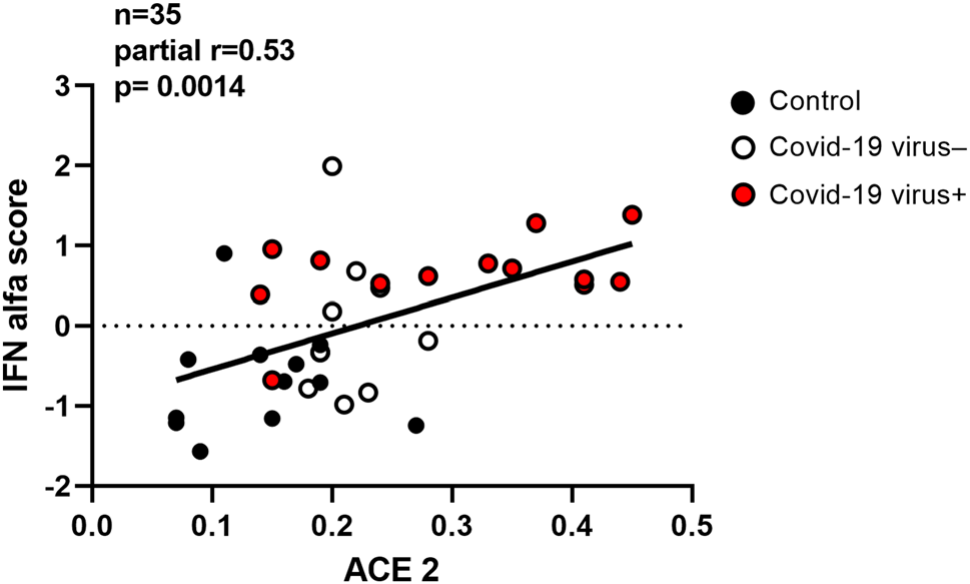
Association of the IFN-alpha score with ACE2 transcription levels after adjustment for age and sex (*n* = 35, partial *R* = 0.53, *p* = 0.0014)

As compared to controls, COVID-19 cases showed no marked deregulations of immune gene transcripts. To highlight possible pathway deregulations in the presence of small fold changes, a GSEA analysis was performed Fig. 2. As compared to the control group, only the IFN-alpha pathway was significantly activated in virus-positive WAT specimens (FDR = 0.009, Fig. 3). SARS-CoV-2 utilizes the ACE2 (and possibly other factors such as BSG, NRP1 and HSPA5) as cell entry factors together with the priming proteases TMPRSS2, CTSL, FURIN that facilitate virus entry [22]. In subcutaneous WAT, only transcripts of ACE2 and FURIN genes could be detected. Transcription of ACE2 was upregulated in COVID-19 cases. Interestingly, the IFN-alpha score was directly related to ACE2 transcripts levels after adjustment for age and sex (*partial* *r* = 0.53, *p* = 0.0014) (Fig. 4). Comparable results were observed also after adjustment for BMI (*partial* *r* = 0.47, *p* = 0.006). Furthermore, transcription of the immunoglobulin alpha Fc receptor (FCAR)) gene was strongly downregulated, suggesting that SARS-CoV-2 plays some immunosuppressive role [23].

## Discussion

Adipose tissue is very abundant in the human body. In adults, the total number of adipocytes in WAT is estimated in about 30 billion with a variable cell size according to age, sex and fat accumulation [24, 25]. The renewal rate is 5–10% per year [26, 27]. The results of this study show that, at the time of death, the SARS-CoV-2 nucleocapsid antigen is expressed in 1 to 5% adipocytes in the virus-positive subcutaneous WAT of COVID-19 cases. In addition, transcripts of the IFN-alpha pathway are upregulated while the transcription levels of ACE2 (but not FURIN and TMPRSS2) are associated with the degree of activation of the IFN-alpha pathway. Notably, expression of PKR (an important marker of IFN activation) was increased in all COVID-19 cases, no matter of SARS-CoV-2 positivity in WAT. The finding indicates that, once triggered, IFN-alpha–a circulating cytokine with a defensive action in uninfected cells—remains active for substantial periods of time stimulating PKR expression [28].

In addition, IFN has been found to stimulate the expression of ACE2 in epithelial cells of the respiratory tract [29] and, more recently, proteomic analyses revealed that in COVID-19 patients, different entry factors of SARS-CoV-2 are comprised in upregulated pathways [30]. Our results show that SARS-CoV-2 infection of WAT is associated with enhanced transcription of ACE2. A condition of this type has been reported in chronic lung diseases that appear to stimulate gene expression programs promoting both the cellular entry of SARS-CoV-2 and the severity of COVID-19 [22]. Comparable mechanisms can be acting in obesity, since the condition is associated with unapparent inflammation [31]. Different WAT-associated endocrine mechanisms have been proposed: (a) high plasma resistin levels are strong predictors of mortality in COVID-19 [32]; (b) high serum leptin levels are associated with increased expression of ACE2 and of leptin receptors in lungs [33]; (c) reduced plasma adiponectin levels associate with respiratory failure in COVID-19 patients (also upon adjustment for age, sex and BMI) indicating a role for adiponectin in the linking of obesity with COVID-19 [34].

Our data also indicate that a sizeable number of adipocytes participate in the production of SARS-CoV-2. As widely reported, virus replication is consistently related to the release of inflammatory factors and cytokines [32, 35, 36]. In COVID-19, a functional ACE2 deficiency has been proposed to cause angiotensin imbalance with the consequent reduction of the ACE2‐RAS system that might worsen the prognosis in obese patients [37]. Regarding the possible immunosuppressive activity of SARS-CoV-2 [36], downregulation of the FCAR gene was the only evidence obtained in our study of WAT. FCAR is expressed in leukocytes, mediates resistance to a wide range of pathogens and stimulates cytokine production [38].

Our study has several limitations. Since observations have been performed on autopsy specimens, the data refer only to the time of death and no information may be given regarding the early or intermediate phases of disease. Also, for safety reasons at the time of autopsy, only subcutaneous WAT was sampled, not visceral WAT, though the latter greatly contributes to overweight and obesity, two recognized risk factors for severe forms of COVID-19. An additional limitation is that—due to autolytic processes in autoptic specimens—the virus could be detected only by immunostaining for the nucleocapsid antigen, not by the more sensitive method of in situ hybridization (ISH) that detects the virus genome [39, 40]. Of note, a previous study of autoptic specimens using ISH and IHC could not detect the virus in adipose tissue and other extra-pulmonary organs [41]. Studies of adipose tissue obtained from living patients would greatly contribute to the field. Also, though this is the first demonstration of SARS COV-2 infection of WAT, the sample size is relatively small. Further studies are needed to clarify the mechanisms underlying the worse clinical outcome in patients with obesity and must include the study of visceral WAT. Lastly, given the relatively high prevalence of cases carrying SARS-CoV-2 in WAT, it should be noted that results refer only to lethal COVID-19 cases. Hence, in less severe cases, virus positivity in WAT cells could be lower.

In conclusion, the data indicate that adipocytes express SARS-CoV-2 protein antigens, that infection activates an IFN response, that the tissue is infiltrated by NK cells, macrophages and T cells. Thus, the adipose tissue directly participates in the disease process and–due to its large size–represents a significant reservoir for virus, an important source of inflammatory mediators, and a possible target for lipophilic drugs.

## Abbreviations

FFPE: Formalin-fixed and paraffin-embedded
BMI: Body mass index (weight in kilograms divided by the square of height in meters)
IFN: Interferon
ACE2: Angiotensin-converting enzyme-2
TMPRSS2: Transmembrane serine protease 2
CTSL: Cathepsin L
PKR: RNA-dependent protein kinase
EIF2S1: Eukaryotic translation initiation factor 2 subunit alpha
FCAR: Immunoglobulin alpha Fc receptor
